# The influence of frequency and temperature on the AC-conductivity in $\text {TlInTe}_2$ semiconductor single crystal

**DOI:** 10.1038/s41598-025-87788-w

**Published:** 2025-02-12

**Authors:** Mohamed M. Fangary, Muhammad A. O. Ahmed

**Affiliations:** 1 Physics Department, Faculty of Science, Luxor University, Luxor, 85951 Egypt; 2 Department of Computer Science, Faculty of Computers and Information, Luxor University, Luxor, 85951 Egypt

**Keywords:** Ac conductivity, Single crystal, Space charge, Thermo gravimetric, Semiconductor, TGA, ML, Ranndome forest, Gradient boosting, Electrical and electronic engineering, Computer science

## Abstract

A special design, based on the Bridgman technique, was used in our laboratory for preparing single crystals of $\text {TlInTe}_2$. The structure of $\text {TlInTe}_2$ in powder form was examined using X-ray diffraction. $\text {TlInTe}_2$ at room temperature was found to be a tetragonal system with lattice parameters of $a = 8.494$ Å and $c = 7.181$ Å. The structural parameters, such as crystallite size *D*, micro strain $\epsilon$, dislocation density $\delta$, and unit cell parameters were determined from XRD spectra. Thermo gravimetric analysis (TGA) was employed to study the thermal behavior of $\text {TlInTe}_2$, showcasing its significance in solid state physics. The TGA curve of $\text {TlInTe}_2$ exhibited distinct weight loss events corresponding to thermal decomposition processes. The frequency and temperature dependence of Ac-conductivity in a $\text {TlInTe}_2$ single crystal was studied by assessing the permittivity ($\epsilon _r$) and dielectric loss ($\tan \delta$) over a broad frequency range. The dependence of AC conductivity and dielectric properties on the frequency and temperature for $\text {TlInTe}_2$ in pellet form obtained from $\text {TlInTe}_2$ single crystal were studied in the frequency range of (40 Hz–3 MHz) and temperature range of $(290{-}395)^{\circ }$K. The AC conductivity of the $\text {TlInTe}_2$ was found to obey the power law, i.e., $\sigma _{ac} (\omega ) = A \omega ^s$. AC conductivity of $\text {TlInTe}_2$ was dominated by the correlated barrier hopping (CBH) model. The obtained activation energy values of the AC conductivity have confirmed that the hopping conduction is the dominant one. A decrease in these values has noticed with the increase in frequency. The density of localized states $N (E_F)$ close to Fermi level for $\text {TlInTe}_2$ was obtained in the range of $(1.02{-}2.8 \times 10^{19}\ \text {eV}^{-1}$ cm$^{-3}$) for various temperatures and frequency. The frequencies corresponding to maxima of the imaginary electric modulus at different temperatures were found to satisfy an Arrhenius law with activation energy $E_R$ of 0.32 eV. A decrease in the relaxation time $\tau$ was observed with the increase in temperature. The average hopping distance *R* and the average time of charge carrier hoping between localized states *t* were found in the range of 6.10–11.95 nm and $2 \times 10^{-7} {-} 2.4 \times 10^{-2}$ s respectively, for the investigated range of frequency and the value of the binding energy $W_m$ was 0.52 eV. We report on the preparation, characterization, and analysis of $\text {TlInTe}_2$ semiconductor single crystals, focusing on the influence of frequency and temperature on AC conductivity. Utilizing X-ray diffraction, thermo gravimetric analysis, and dielectric property measurements, we delineate the material’s structural and electrical properties. Complementing our experimental findings, Machine Learning (ML) models, including Random Forest and Gradient Boosting, were employed to predict AC conductivity, revealing significant predictors and corroborating the experimental insights with high accuracy. This interdisciplinary approach enhances our understanding of $\text {TlInTe}_2$’s properties and demonstrates the potential of ML in materials science research.

## Introduction

The ternary *TlMeX*(*MeX* : *InSe*2, *InTe*2...) compounds have been attractive due to their unusual electronic, optoelectronic devices, chain structure, and physical properties^1^. $\text {TlInTe}_2$ belongs to the group of chalcogenides with a chain crystal structure often called in literature as TlTe-type one. This compound crystallizes in a tetragonal body-centered lattice and its chemical composition can be presented as $\hbox {Tl}^{+1}(\hbox {In}^{+3}\hbox {Te}^{-2})$^2^. The performance of various electrical depends on the properties of dielectric materials. Dielectric properties are affected by pressure, temperature, and frequency. The study of such properties is, in particular, a fascinating physical properties^3^. $\text {TlInTe}_2$ is another attractive chain structured compound and thought as fascinating compound for efficient thermoelectrics and thermal barrier coatings due to its ultralow lattice thermal conductivity^4^. A semiconductor detector of neutron radiation based on a $\text {TlInTe}_2$ crystal has been investigated^5^. $\text {TlInTe}_2$ crystals were used to design radio frequency sensitive varactor device^6^. Some of the physical properties of $\text {TlInTe}_2$ crystal system such as specific heat capacities^7^, optical and electrical properties^8,9^, dielectric measurements, and thermoelectric power^10^ have been published. $\text {TlInTe}_2$ crystals belong to the tetragonal system [space group *I*4/*mcm* (140)] with the unit cell parameters $a = 8.494$  Å and $c = 7.181$  Å^11^. Studies on the temperature and frequency dependence of the Ac conductivity of  $\text {TlInTe}_2$ crystals revealed that the Ac conductivity of   $\text {TlInTe}_2$ is dominated by the CBH model^12^. AC conductivity measurements of semiconductors are an important tool to understand the conduction mechanisms in these materials^13^. Various models, such as quantum-mechanical tunneling (QMT) model^14,15^, large polar on tunneling (LPT) model^16^, small polar on tunneling (SPT) model^17^, and CBH model^18,19^, have been proposed to explain the AC conduction mechanism in different materials. The primary goal of this study is to investigate the frequency and temperature dependence of AC conductivity in $\text {TlInTe}_2$ single crystals, focusing on elucidating the underlying charge transport mechanisms. By leveraging experimental techniques such as X-ray diffraction and thermo-gravimetric analysis, we analyze the material’s structural and dielectric properties in detail. Particular attention is given to the role of the CBH model in explaining the observed conduction behavior. To further enhance our understanding, ML models, including Random Forest and Gradient Boosting, are employed to predict AC conductivity based on experimental data alongside CBH. This interdisciplinary approach integrates experimental insights with computational methods, providing a comprehensive understanding of $\text {TlInTe}_2$’s potential for advanced electronic and optoelectronic applications.

## Related work

Recent advancements in computational materials science have seen the successful integration of ML models to predict various material properties, from electronic band structures to thermal conductivity. The authors in^20^ used Raman spectroscopy, X-ray diffraction, and transport measurements. They combined experiments with theoretical crystal structure predictions. The study found a transition from a semiconductor to a semimetal at 4 GPa. A superconducting phase appeared at 5.7 GPa. An unusual phonon mode softening was observed at higher pressures. The structural studies showed no phase transitions up to 33.5 GPa. However, new high-pressure phases were predicted above 35 GPa. The work highlights the role of electron-phonon coupling and Lifshitz transitions in $\text {TlInTe}_2$. It opens new possibilities for studying pressure-induced phenomena in similar materials In exploring the intersection of ML and semiconductor technologies, Liu et al.^21^ elucidate on the burgeoning role of ML in advancing semiconductor material science and manufacturing. The study outlines the crucial aspects of data preparation, feature engineering, and the application of various ML algorithms to address challenges in semiconductor research. Significantly, it delves into ML’s contribution to new material discovery, property prediction, and the optimization of manufacturing processes, underscoring the dynamic evolution and future potential of ML applications within the semiconductor industry. Huang et al. contribute to the field by highlighting the crucial role of ML in accelerating material innovation. They explore ML’s efficiency in reducing computational costs and development cycles for new materials, covering applications in areas like superconductivity and photovoltaics. The review outlines basic ML principles, discusses commonly used algorithms, and examines their role in predicting material properties and aiding synthesis, pointing towards a promising future for ML in materials science^22^. The review by Mobarak et al. delves into ML’s broad applications within materials research, spanning six crucial dimensions and redefining the field’s scope. It elucidates on diverse ML techniques from supervised to deep learning, showcasing their transformative impact on material selection, structure-property relationship understanding, and innovative discovery. The review also highlights ML’s advancements in image processing and its pivotal role at the atomic level, offering a comprehensive view of how ML propels materials research forward, despite challenges such as data quality and complex algorithms^23^. Karande, Gallagher, and Han provide a strategic guide for material scientists embarking on ML projects, addressing the critical steps from problem formulation to real-world application. They emphasize the importance of domain knowledge, data curation, feature representation, model selection, and ensuring model generalizability. Through a case study on predicting the compressive strength of TATB samples, they illustrate the impact of methodical decision-making in ML pipelines, aiming to enhance the reliability of ML predictions in material science^24^.

Recent advancements in ML have profoundly impacted the semiconductor industry, revolutionizing various aspects of design and manufacturing processes. This section explores significant contributions in ML-based defect classification, data analytics for semiconductor scaling, and the evaluation of semiconductor quantum dots^25^. One notable study by Taha (2024) offers a comprehensive review of ML techniques for identifying wafer defects in semiconductor manufacturing. This research consolidates existing literature, highlighting the strengths and limitations of various ML algorithms used in defect detection. Taha introduces a novel taxonomy that categorizes these methodologies, providing a structured framework to understand the complex relationships between different algorithms. The study underscores the potential for future advancements in ML classification techniques, which are crucial for enhancing wafer defect detection and improving overall manufacturing quality^26^. In another significant contribution, an article by SPIE (2024) delves into the use of advanced data analytics and ML methodologies for continued semiconductor scaling. The study discusses the application of topological data analysis techniques to optimize physical design, manufacturing processes, and IC product yield. By representing physical domain space as a high-dimensional topological network, the researchers demonstrate how ML can enhance the identification of yield detractors and optimize design-process-yield relationships. This innovative approach bridges the gap between empirical observations and algorithmic predictions, offering a robust framework for improving semiconductor design and manufacturability^27^.

## Experimental technique

In order to grow perfect TlInTe$_2$ single crystals, a modified Bridgman-Stockbarger technique was used. The ampoule was charged with a required amount of material: $24.9095\,g$ of pure thallium representing $35.585\%$ (Aldrich mark) $99.9999\%$, $13.9902\,\hbox {g}$ of pure indium representing $19.986\%$ (Aldrich mark) $99.9999\%$, and $31.1003\,\hbox {g}$ of pure tellurium representing $44.429\%$. The appropriate amount was first sealed in a silica ampoule at a pressure of $10^{-5}\, \text {Torr}$. At the beginning of the growth run, the ampoule was held in the hot zone of the furnace at $1145\,\hbox {K}$ for about 24 h for melt homogenization. Then, the melt was shaken during heating several times to accelerate the diffusion of the constituents through each other. The mechanical system is always used to draw the charged ampoule from one zone to another with the required rate; in our case, the charged ampoule is lowered gradually and slowly through temperature steps at a rate of about $1.6\,\hbox {mm/h}$. The temperature of the middle zone is $1050\,\hbox {K}$, corresponding to the crystallization temperature of TlInTe$_2$ according to the phase diagram^28^. The duration time for producing TlInTe$_2$ as a single crystal is about 12 days. The powder of TlInTe$_2$ was obtained from single crystals cleaved, which was ground finally and compressed under a suitable pressure to form a pellet of about $9\,\hbox {mm}$ diameter and $1.35\,\hbox {mm}$ thickness. X-ray diffraction (XRD) technique was utilized to identify the crystalline nature of the powder of TlInTe$_2$ at room temperature. For this purpose, a Philips X-ray diffractometer (model X’Pert) was used for the measurements, with monochromatic Cu ($K\alpha$), operated at $40\,\hbox {KV}$ and $25\,\hbox {mA}$. The TGA was conducted using a state-of-the-art thermogravimetric analyzer. Approximately $10\,\hbox {mg}$ of finely powdered TlInTe$_2$ sample was placed in a platinum crucible. The sample was then heated from room temperature to $800\,^{\circ }\hbox {C}$ at a rate of $10\,^{\circ }\hbox {C/min}$ under a nitrogen atmosphere to prevent oxidation. The ohmic contacts were achieved by two (Au) electrodes on the parallel surfaces of the sample. For dielectric properties and AC conductivity measurements, the sample holder was inserted into an electric furnace. The AC conductivity and dielectric measurements were carried out on this sample using a programmable automatic RLC bridge (model Hioki 3532 Hitester). The impedance *Z*, the capacitance *C*, and the loss tangent $\tan \delta$ were directly measured in the frequency range 40 Hz–3 MHz. The temperature of the sample was measured by using a Chromel-Alumel thermocouple in the range from 295 to 395 K. The AC conductivity $\sigma _{\text {ac}}(\omega )$ of bulk TlInTe$_2$ is determined by the following relation:

1 $$\begin{aligned} \sigma _{\text {ac}}(\omega ) = \sigma _{\text {tot}}(\omega ) - \sigma _{\text {dc}}(\omega \rightarrow 0), \end{aligned}$$

where $\sigma _{\text {tot}}(\omega )$ is the total conductivity calculated using the formula:

2 $$\begin{aligned} \sigma _{\text {tot}}(\omega ) = \frac{d}{A Z}, \end{aligned}$$

where *A* is the cross-sectional area of the pellet with a value of $(7.85 \times 10^{-5}\,\hbox {m}^2)$, *d* is the thickness. The permittivity $\varepsilon _1$ of the material was calculated using the formula:

3 $$\begin{aligned} \varepsilon _1 = \frac{C d}{\varepsilon _0 A}, \end{aligned}$$

where $\varepsilon _0$ is the permittivity of free space. As well, the dielectric loss $\varepsilon _2$ was calculated using the relation:

4 $$\begin{aligned} \varepsilon _2 = \varepsilon _1 \tan \delta , \end{aligned}$$

where $\delta = 90\,^{\circ } - \Phi$ and $\Phi$ is the phase angle. The AC conductivity of the sample ($\sigma _{\text {ac}}$) was determined from dielectric parameters using this relation:

5 $$\begin{aligned} \sigma _{\text {ac}} = \omega \varepsilon _0 \varepsilon _2, \end{aligned}$$

where $\omega$ is the angular frequency.

**Fig. 1:**
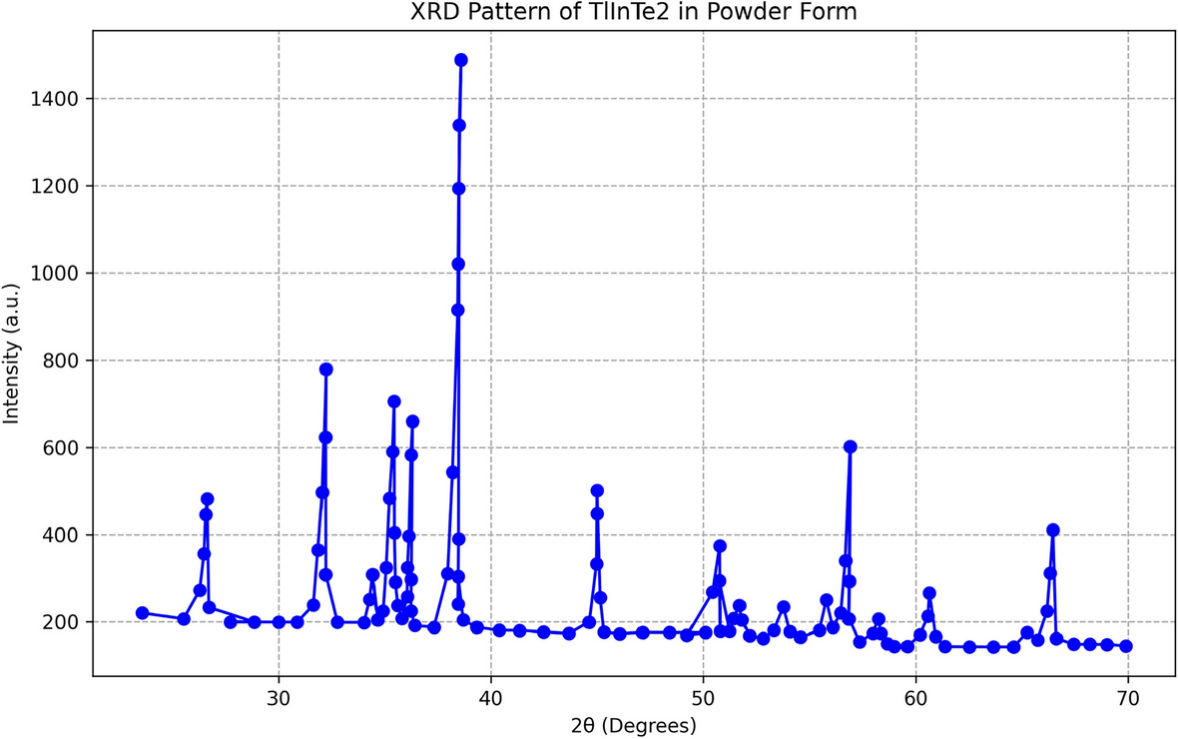
XRD pattern of TlInTe$_2$ in powder form.

**Fig. 2:**
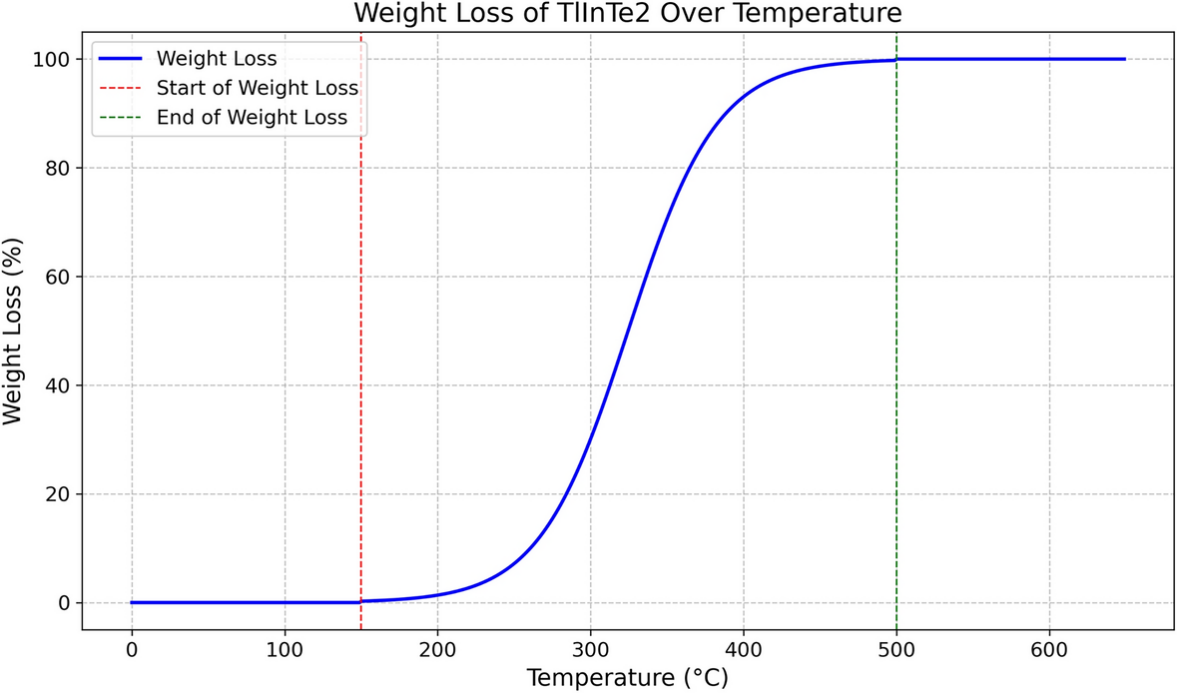
weight loss of Thallium Indium Telluride ($\text {TlInTe}_2$) over a temperature.

## Results and discussion

The X-ray diffraction (XRD) pattern of TlInTe$_2$ in powder form at room temperature is shown in Fig. 1. As observed, the powder XRD pattern has revealed a polycrystalline nature of the investigated sample. The data analysis of the structure was indexed using (JCPDS card; number 71-2195). The analysis is matched with tetragonal structure with lattice parameters $a = 8.494$ Å, $b= 8.494$ Å, and $c = 7.181$ Å which in accordance with k.Wakita et al^29^. The highest preferred orientation is found along the (202) plane. The crystallite size *D* of the bulk TlInTe$_2$ was calculated according to the following equation^30^:

6 $$\begin{aligned} D = k \frac{\lambda }{\beta \cos \theta } \end{aligned}$$

where *k* is the shape factor which equals 0.95, $\lambda$ is the wavelength of Cu ($K\alpha$) radiation, $\beta$ is the experimental observed peak width at half maximum height (FWHM), and $\theta$ is Bragg’s angle. The dislocation density $\delta$ was determined by the following equation^31^:

7 $$\begin{aligned} \delta = \frac{1}{D^2} \end{aligned}$$

The micro strain $\epsilon$ developed in the prepared TlInTe$_2$ pellet was calculated by using the following relation:

8 $$\begin{aligned} \epsilon = \frac{\beta \cot \theta }{4} \end{aligned}$$

The structural parameters of TlInTe$_2$ are listed in Table 1.

**Table 1:** Structural parameters of TlInTe$_2$.

(hkl)	Relative intensity (%)	*D* (nm)	$\delta$ ($\times 10^{-4}$ nm$^{-2}$)	$\epsilon$ ($\times 10^{-3}$)
(202)	100.00	58.06	2.97	2.33
(200)	45.41	62.19	2.17	2.42
(420)	26.14	71.28	1.97	1.24

Figure 2 shows the TGA curve of TlInTe$_2$. The TGA curve of TlInTe$_2$ revealed several key thermal events:

- Initial Weight Loss (50–150$\,^{\circ }$C): Attributed to the desorption of surface-bound water and volatile contaminants. This phase is minor, with a weight loss of less than 1%.
- Stable Phase (150–500$\,^{\circ }$C): No significant weight loss was observed, indicating high thermal stability in this temperature range, crucial for semiconductor applications.
- Onset of Decomposition ($>\,500\,^{\circ }$C): A marked weight loss was observed, suggesting the commencement of thermal decomposition. This phase is characterized by the breakdown of the TlInTe$_2$ lattice, leading to the formation of simpler tellurium, indium, and thallium compounds.

The thermal stability of TlInTe$_2$ up to 500 $^{\circ }$C is promising for various applications, including optoelectronics and thermal barrier coatings. The onset of decomposition at higher temperatures delineates the operational boundaries and aids in material selection for specific applications.

### Temperature and frequency dependencies of AC electrical conductivity

#### Temperature dependence of AC electrical conductivity

**Fig. 3:**
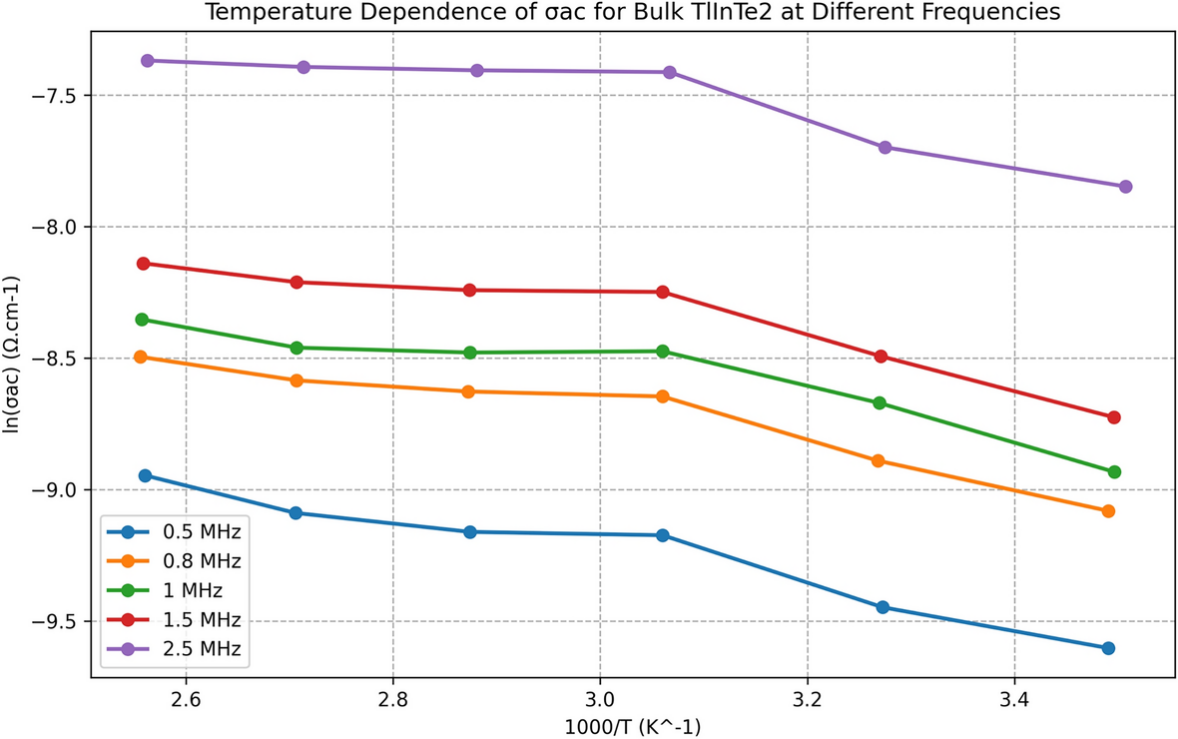
Temperature dependence of $\sigma _{\text {ac}}$ for bulk TlInTe$_2$ at different frequencies.

Figure 3 shows the disparity of AC electrical conductivity with temperature for bulk TlInTe$_2$ at different frequencies =[0.5, 1, 1.5, 2.5, 3] MHZ. The figure shows a semiconductor-like behavior in the full range of temperature with a linear relationship between $\ln (\sigma _{\text {ac}})$ and the inverse of temperature at different frequencies. The increase in conductivity with the rising temperature is owing to the increase in the thermally activated electron drift velocity of charge carriers in accordance with the hopping conduction mechanism. This indicates that $\sigma _{\text {ac}}$ is a thermally activated process^32^.

**Fig. 4:**
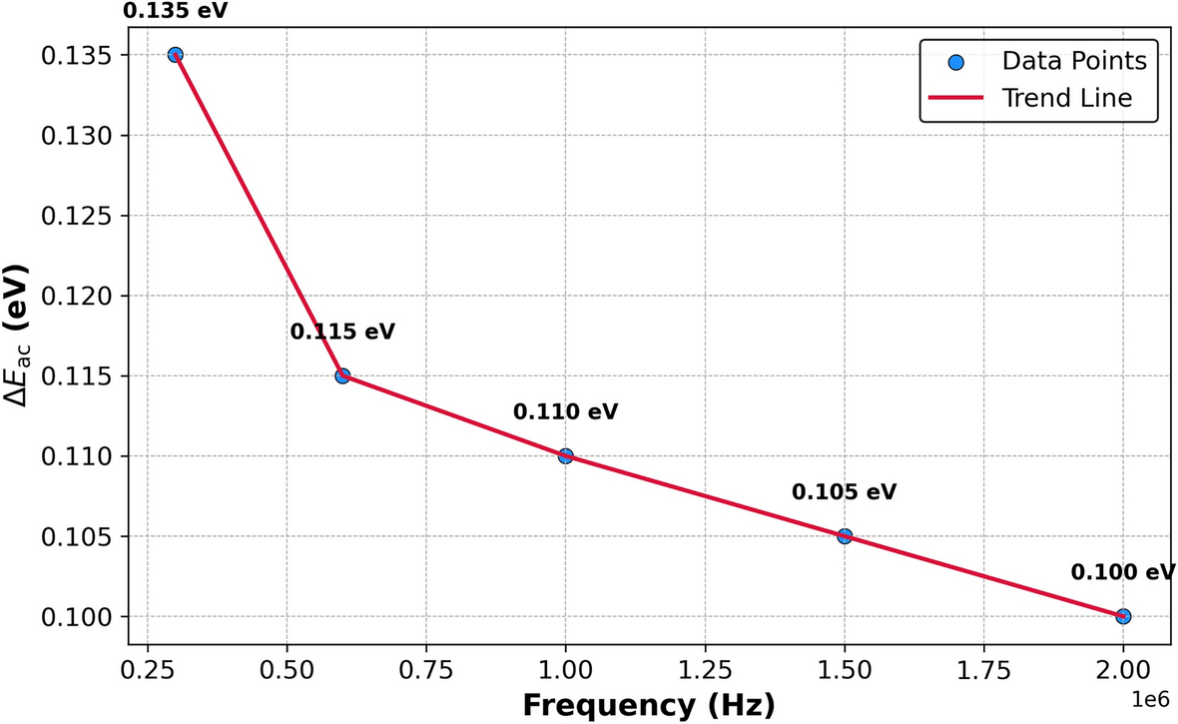
Illustares the variation of $\Delta E_{\text {ac}}$ for bulk TlInTe$_2$ at different frequencies.

From Fig. 3, we calculate the activation energy $\Delta E_{\text {ac}}$ of the AC using the normal Arrhenius equation:

9 $$\begin{aligned} \sigma _{\text {ac}} = \sigma _0 \exp \left( -\frac{\Delta E_{\text {ac}}}{k_BT}\right) \end{aligned}$$

where $\sigma _0$ is the pre-exponential constant. The values of $\Delta E_{\text {ac}}$ for different frequencies are shown in Fig. 4.

It is observed that $\Delta E_{\text {ac}}$ decreases with increasing the applied frequency. The decrease of $\Delta E_{\text {ac}}(f)$ is due to the increase of the applied field which reinforces the jump of charge carriers between the localized states; accordingly, the activation energy decreases with the increase of frequency. Such a decrease emphasizes that hopping conduction is the dominant mechanism.

#### Frequency dependence of AC electrical conductivity

The dependence of AC conductivity, $\sigma _{\text {ac}}$, on the frequency of TlInTe$_2$ as shown in Fig. 4 at different temperatures. It is observed in Fig. 4 that above a certain point, the AC conductivity ($\sigma _{\text {ac}}$) increases linearly with frequency, and the DC contribution is important at lower frequencies and high temperatures, whereas the frequency-dependent term dominates at higher frequencies. In the low-frequency region, the electrical conductivity depends on temperature. The dependence of AC conductivity on the frequency of the applied field is given by the following equation^33^

10 $$\begin{aligned} \sigma _{\text {Ac}}(\omega ) = A\omega ^s \end{aligned}$$

where *A* is a constant dependent on temperature, $\omega$ is the angular frequency, and *s* is the frequency exponent which generally is less than or equal to one. The value of the frequency exponent *s* can be determined from the slope of the linear parts in Fig. 4 at different temperatures.

**Fig. 5:**
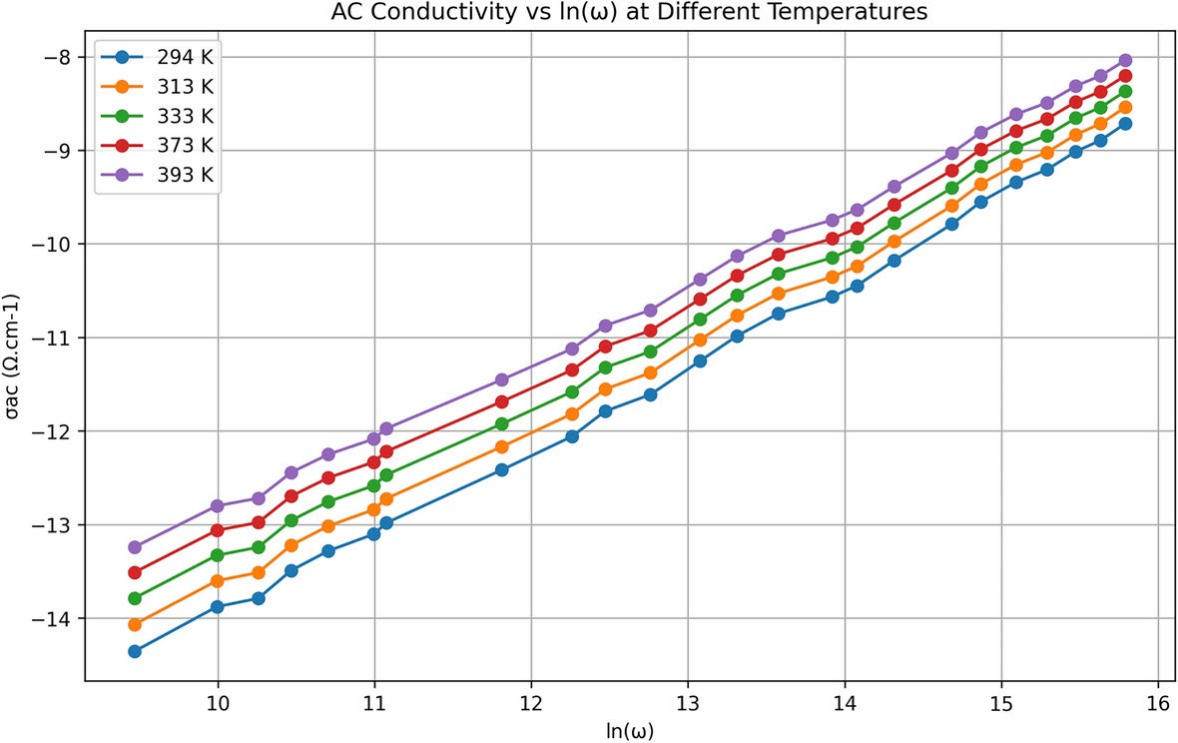
Frequency dependence of $\sigma _{\text {ac}}(\omega )$ of bulk TlInTe$_2$ at different temperatures.

The value of exponent *s* and its behavior with temperature or frequency determine the dominant conduction mechanism. It is noticed that *s* value decreases with the increase of temperature as observed in the inset of Fig. 5. The dependence of *s* on temperature and its range of values for TlInTe$_2$ are consistent with the CBH model. Thus, the mechanism of charge transport in this frequency range for TlInTe$_2$ is a hopping mechanism over localized states at near the Fermi level, because the CBH model^34^ predicts *s* to be both temperature and frequency-dependent and *s* should decrease with increasing temperature, which is in agreement with the findings reported by Sheleg et al.^12^. According to the CBH model, the magnitude of the binding energy $W_m$ of the carrier in its localized sites, which is related to the maximum barrier height at infinite intersite separation, is determined from the equation of frequency exponent *s*^35^.

11 $$\begin{aligned} s = 1 - \frac{6k_BT}{W_m - k_BT \ln (\omega \tau _0)} \end{aligned}$$

where $\tau _0$ is the characteristic relaxation time. For a large value of $\frac{W_m}{k_BT}$, the exponent *s* is near unity. Also, Eq. (6) predicts that *s* decreases with increasing temperature at large $\frac{W_m}{k_BT}$. The above demonstrations lead to the following equation^36^:

12 $$\begin{aligned} s = 1 - \frac{6k_BT}{W_m} \end{aligned}$$

**Fig. 6:**
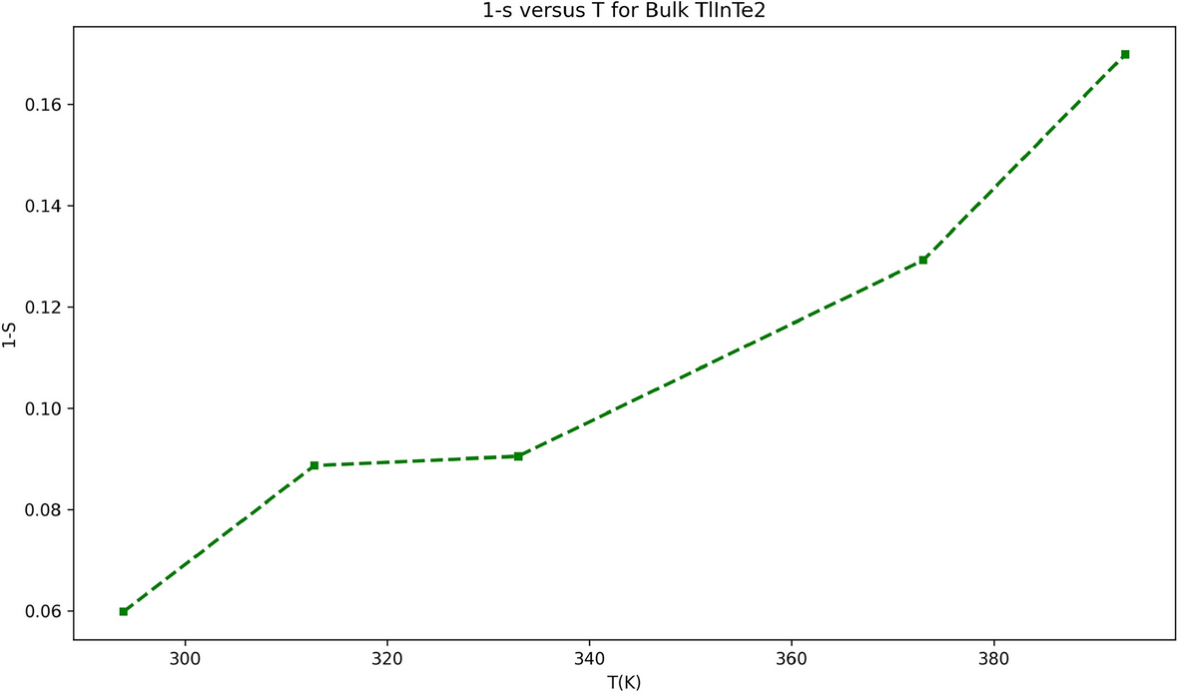
Plot of $1 - s$ versus *T* for bulk TlInTe$_2$.

To calculate the binding energy $W_m$, the values of $(1 - s)$ were plotted versus temperature *T* and are shown in Fig. 5. And the binding energy $W_m$ was determined from the slope of Fig. 6 to be $0.52\,\text {eV}$.

According to the Austin–Mott formula^37^, based on the CBH model, the AC conductivity $\sigma _{\text {ac}}(\omega )$ can be interpreted as hopping of electrons between a pair of localized states near the Fermi level, $E_F$. Therefore, the process of hopping of electrons is influenced by the density of localized states $N(E_F)$ close to the Fermi level. $\sigma _{\text {ac}}(\omega )$ is related to the number of sites per unit energy per unit volume $N(E_F)$ at the Fermi level as follows:

13 $$\begin{aligned} \sigma _{\text {ac}}(\omega ) = \left( \frac{\pi }{3}\right) [N(E_F)]^2 k_B Te^2 \alpha ^{-5} \omega [\ln (\upsilon _p / \omega )]^4 \end{aligned}$$

where *e* is the electronic charge, $k_B$ is the Boltzmann’s constant, $\alpha$ is the exponential decay parameter of localized states wave functions, and $\upsilon _p$ is the frequency of the phonons. By assuming $\upsilon _p = 10^{12}\,\hbox {s}^{-1}$ and $\alpha ^{-1} = 10$ Å, $N(E_F)$ was calculated at different frequencies and temperatures. The values of $N(E_F)$ were found to be in the range of $(1.02-2.8 \times 10^{19}\,\text {eV}^{-1}\,\text {cm}^{-3})$ for the investigated range of temperature and frequency.

According to the theory of AC hopping conductivity, we could determine the average time *t* of charge carriers hopping from one localized state to another and the average hopping distance *R* using the following equations^38^:

14 $$\begin{aligned} t^{-1}= & \upsilon _P \exp (-2R\alpha ) \end{aligned}$$

15 $$\begin{aligned} R= & \left( \frac{1}{2\alpha }\right) \ln (\upsilon _P / f) \end{aligned}$$

The values of *t* and *R*, depending on the investigated frequency range, were found in the range of $(2 \times 10^{-7}-2.4 \times 10^{-2}\,\hbox {s})$ and $(6.10{-}11.95\,\hbox {nm})$, respectively. The values of $N(E_F)$, *t*, and *R* for TlInS$_2$ single crystals at $T = 297\,K$ and $f = 2.55 \times 10^5\,\hbox {Hz}$ have been reported by El-Nahass et al.^39^, and found as: $1.5 \times 10^{20}\,\text {eV}^{-1}\,\text {cm}^{-3}$, $3.79\,\upmu \hbox {s}$, and 6.07 nm, respectively. While for TlGaS$_2$ single crystals, their values were reported as: $2.1 \times 10^{18}\,\text {eV}^{-1}\,\text {cm}^{-3}$, $2\,\upmu \hbox {s}$, and $10.3\,\hbox {nm}$, respectively.

### Thermo gravimetric analysis (TGA)

The TGA curve of $\text {TlInTe}_2$ exhibited distinct weight loss events corresponding to thermal decomposition processes. Initial weight loss around 50–150 $^{\circ }$C was attributed to the desorption of surface-bound water and volatile contaminants.

### AC electrical conductivity

The temperature dependence of AC electrical conductivity showed a semiconductor-like behavior with a linear relationship between $\ln (\sigma _{ac})$ and the inverse of temperature at different frequencies. The activation energy $\Delta E_{ac}$ for different frequencies was calculated using the Arrhenius equation:

$$\begin{aligned} \sigma _{ac} = \sigma _0 \exp \left( -\frac{\Delta E_{ac}}{k_B T}\right) \end{aligned}$$

where $\sigma _0$ is the pre-exponential constant. In addition to the observed semiconductor-like behavior, the activation energy values calculated for each frequency were analyzed and compared with literature, indicating consistency with the CBH model. A discussion of prefactor values ($\sigma _0$) is provided to contextualize these results with previous findings.

**Fig. 7:**
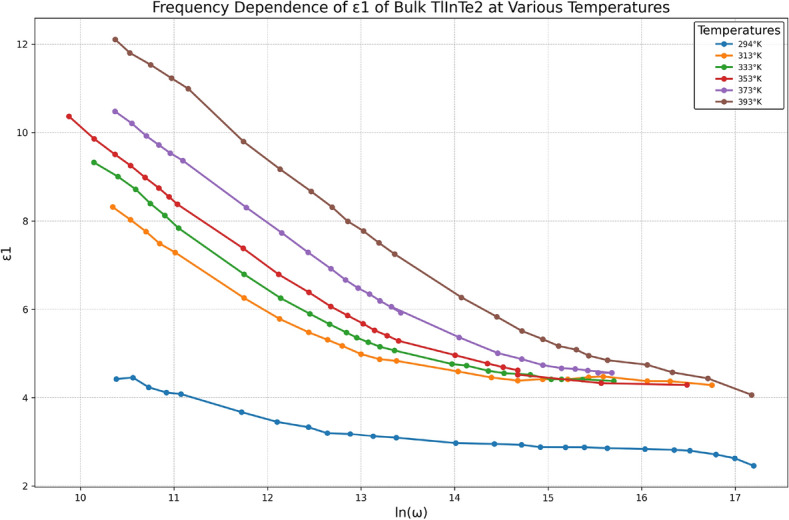
Frequency dependence of $\varepsilon _1$ of bulk $\text {TlInTe}_2$ at various temperatures.

### Dielectric properties

Dielectric relaxation studies are important to understand the nature and the origin of dielectric losses, which in turn, may be useful in the determination of the structure and defects in solids. When an electric field is applied to any matter, the latter dissipates a certain quantity of electric energy that turns into heat energy. This phenomenon is commonly known as loss of power, meaning an average electrical power dissipated in matter during a certain interval of time. The amount of power losses in a dielectric under the action of the applied field is commonly known as dielectric losses. The complex dielectric function $\varepsilon ^*(\omega )$ is expressed as:

16 $$\begin{aligned} \varepsilon ^*(\omega ) = \varepsilon _1(\omega ) + i\varepsilon _2(\omega ) \end{aligned}$$

where $\varepsilon _1(\omega )$ is the real part of the complex permittivity which corresponds to the permittivity of the sample, and $\varepsilon _2(\omega )$ is the imaginary part of the complex permittivity, which corresponds to the dielectric loss used to determine the type of dielectric mechanism of the sample. The frequency dependence of the permittivity $\varepsilon _1(\omega )$ and dielectric loss $\varepsilon _2(\omega )$ for bulk TlInTe$_2$ at different temperatures are revealed in Fig. 7.

**Fig. 8:**
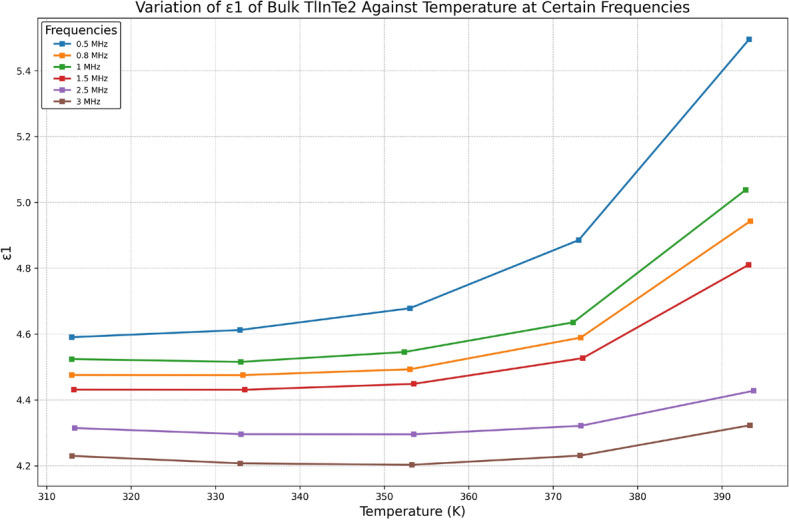
Frequency dependence of the permittivity, $\varepsilon _1$, of bulk TlInTe$_2$ at various temperatures.

Figure 8 shows that $\varepsilon _1(\omega )$ decreases with increasing frequency. The decrease of $\varepsilon _1(\omega )$ with frequency can be explained as follows: at low frequencies, $\varepsilon _1(\omega )$ is owing to the contribution of multicomponent polarization (deformational polarization and relaxation polarization). When the frequency is increased, the dipoles will no longer be able to rotate sufficiently rapidly, so that their oscillation will lag behind those of the field. As the frequency increases, the dipoles will be completely unable to follow the field, and the orientation polarization vanishes; hence, $\varepsilon _1(\omega )$ starts to decrease, moving towards a constant value at a high frequency due to the space charge polarization^40^.

**Fig. 9:**
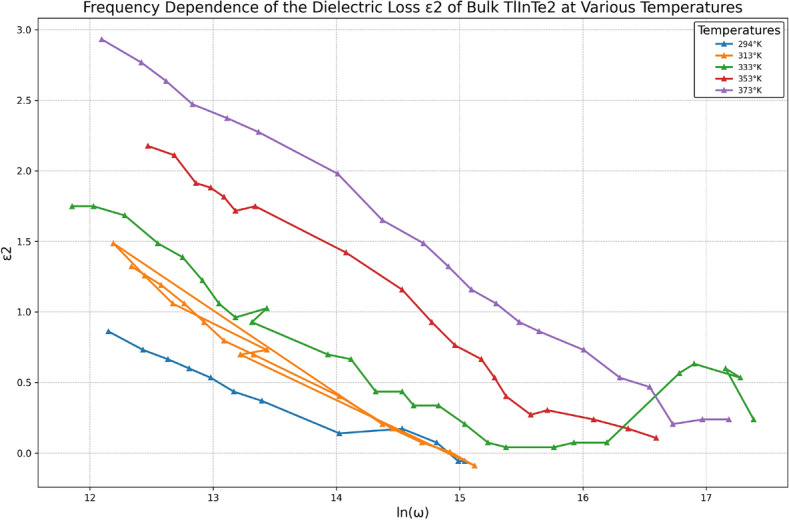
Frequency dependence of the dielectric loss $\varepsilon _2$ of bulk TlInTe$_2$ at various temperatures.

Figure 8 shows that $\varepsilon _1(\omega )$ increases with increasing temperature over the whole range of frequency investigated. The increase of $\varepsilon _1$ can be ascribed to the fact that the dipoles cannot orient themselves at low temperature. With increasing temperature, the orientation of dipoles is facilitated and the values of the orientational polarization are increased, which leads to an increase in the permittivity $\varepsilon _1$^40^.

**Fig. 10:**
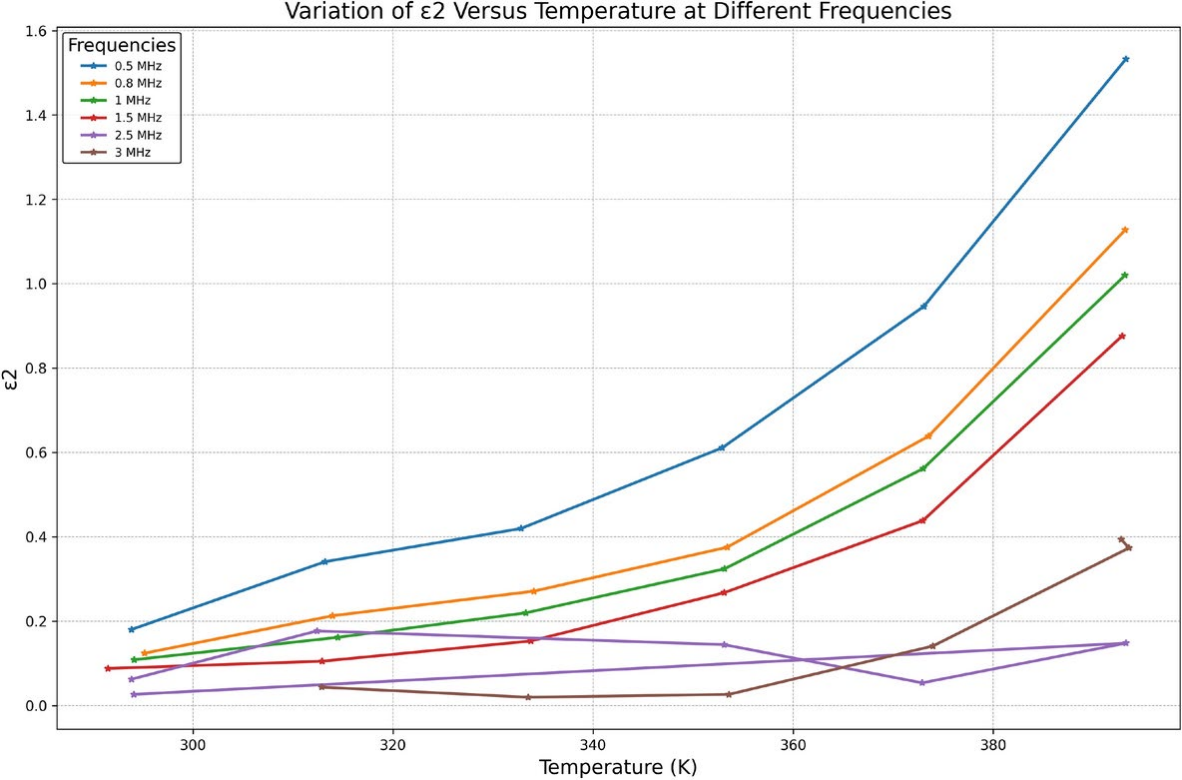
Variation of $\varepsilon _2$ versus temperature at different frequencies.

The dependence of the dielectric loss $\varepsilon _2$ on the frequency is plotted at different temperatures as shown in Fig. 9. It is observed that the behavior of $\varepsilon _2(\omega )$ is similar to $\varepsilon _1(\omega )$, i.e., decrease with increasing frequency. Also, Fig. 9 shows that $\varepsilon _2$ exhibits strong temperature dependence at low frequency. As the temperature increases, the electrical conduction losses increase, which increases the values of the dielectric loss, $\varepsilon _2$, as can be seen in Fig. 10. It is noticed that the temperature dependence of the permittivity of TlInTe$_2$ demonstrates the absence of anomalies in the range of temperature studied; consequently, the absence of phase transitions in this range.

**Fig. 11:**
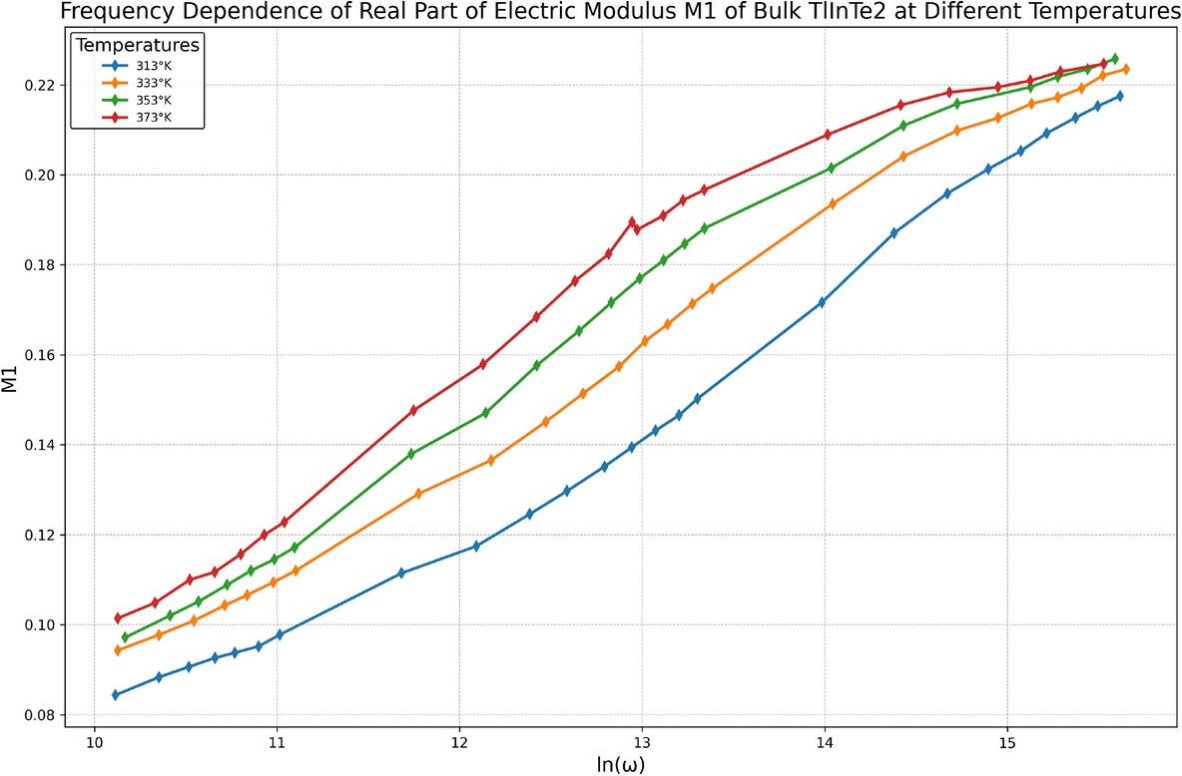
Frequency Dependence of Real Part of Electric Modulus M1 of Bulk $\text {TlInTe}_2$ at Different Temperatures.

### Electric modulus analysis

Dielectric relaxation studies can be explained by the dielectric modulus representation that in the absence of a well-defined $\varepsilon _2(\omega )$ peak^41^. From the physical perspective, the electrical modulus agrees with the relaxation of the electric field in the materials when the electric displacement remains constant. The complex dielectric modulus $M^*(\omega )$ is described by^42^:

17 $$\begin{aligned} M^*(\omega ) = \frac{1}{\varepsilon ^*(\omega )} = \frac{1}{\varepsilon _1(\omega ) + i\varepsilon _2(\omega )}; \quad M^*(\omega ) = M_1 + iM_2 \end{aligned}$$

And

18 $$\begin{aligned} M_1 = \frac{\varepsilon _1}{\varepsilon _1^2+\varepsilon _2^2}, \quad M_2 = \frac{\varepsilon _2}{\varepsilon _1^2+\varepsilon _2^2} \end{aligned}$$

**Fig. 12:**
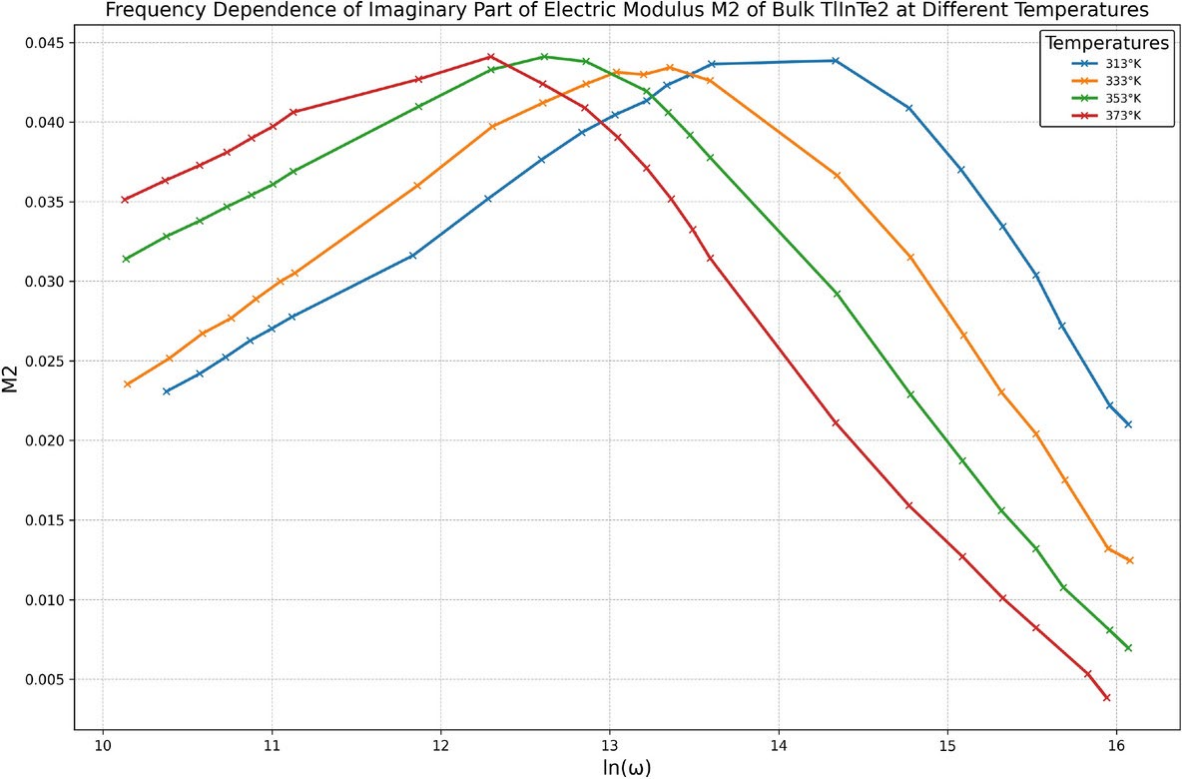
Frequency dependence of the imaginary part of electric modulus $M_2$ of bulk TlInTe$_2$ at different temperatures.

Where $\varepsilon ^*(\omega )$ is the complex dielectric permittivity. Based on Eq. (11), the presentation of the dielectric data was changed from $\varepsilon _1(\omega )$ and $\varepsilon _2(\omega )$ to $M_1(\omega )$ and $M_2(\omega )$. The obtained modulus spectra $M_1(\omega )$ and $M_2(\omega )$ are drawn in Figs. 11 and 12 respectively.

**Fig. 13:**
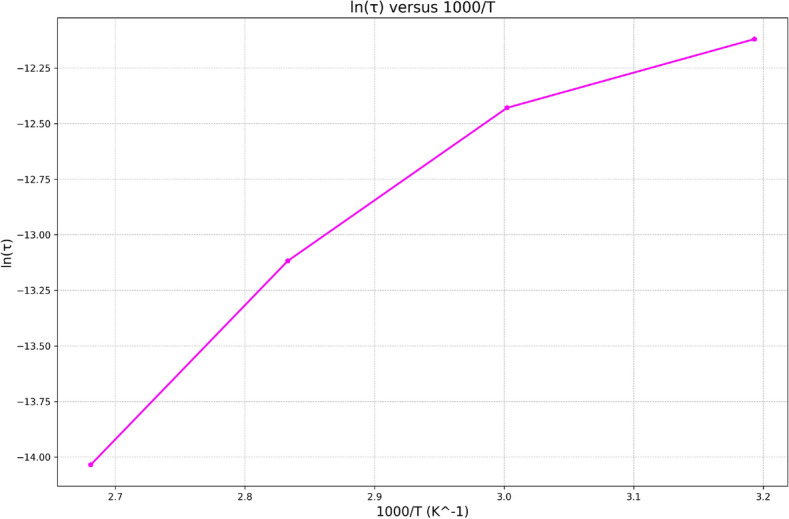
Plot of $\ln (\tau )$ versus 1000/*T*.

The presentation of data by this method showed an obvious relaxation peak for $M_2(\omega )$ that moves toward higher frequencies with increasing temperature revealing the thermally activated nature of the relaxation time $\tau$. The frequency region below peak maximum determines the range in which charge carriers are mobile on long range distance, and above peak maximum, the carriers are confined to potential wells being mobile on short distances. As an appropriate measure of the characteristic relaxation time $\tau$, one can choose the inverse of the frequency of the maximum peak position, i.e., $\tau = \omega _m^{-1}$. Thus, we can determine the dependence of $\tau$ on the temperature as shown in Fig. 13, which follows Arrhenius law^43^:

19 $$\begin{aligned} \tau = \tau _{\infty } \exp \left( \frac{E_R}{k_B T}\right) \end{aligned}$$

Where $E_R$ is the activation energy of the relaxation process, and $\tau _{\infty }$ is the relaxation time at infinite temperature. Values of $E_R$ and $\tau _{\infty }$ were calculated from the slope and intercept of the linear variation of $\ln (\tau )$ versus 1000/*T*, which equal 0.32 eV and $4.6 \times 10^{-11}\,\hbox {s}$ respectively.

The decrease in the relaxation time is due to the dipoles following the motion of the alternating field owing to the dissipation of thermal energy.

## Incorporation of ML insights into AC electrical conductivity analysis

The integration of ML and physics provides unique insights into the underlying material properties. While ML excels at uncovering patterns in high-dimensional data, it depends heavily on data quality and precision. This reliance underscores the importance of accurate experimental measurements. For instance, discrepancies in measured dielectric properties can significantly affect ML predictions, leading to potential misinterpretations of physical models. Understanding such nuances is crucial for the effective application of ML in materials science. In this study, ML was used to complement experimental analysis and the CBH model. ML allowed us to predict AC conductivity and understand its relationship with material properties such as temperature, frequency, and structural parameters. The goal was to use ML to optimize the synthesis of $\text {TlInTe}_2$ crystals and validate experimental findings. This approach ensures consistency between theoretical, computational, and experimental results.

### Synthetic dataset and features

We generated a synthetic dataset of 10,000 samples based on experimental ranges and theoretical principles. Each sample included the following features:

- **Temperature (K)**: 150 to 600 K.
- **Frequency (Hz)**: 1 to 5 MHz.
- **Crystallite Size (nm)**: 40 to 80 nm.
- **Dislocation Density (1/m**$^{\textbf{2}})$: $1 \times 10^{14}$ to $3 \times 10^{14}$.
- **Micro Strain**: 0.001 to 0.003.
- **permittivity**: 0.85 to 6.
- **AC Conductivity (S/m)**: Calculated using:

20 $$\begin{aligned} \sigma _{AC} = \epsilon _0 \epsilon _r \omega \tan (\delta ) \end{aligned}$$

Here, $\epsilon _0$ is the permittivity of free space ($8.854 \times 10^{-12}$ F/m), $\epsilon _r$ is the permittivity, $\omega$ is the angular frequency ($2\pi f$), and $\tan (\delta )$ is the loss tangent, assumed constant at 0.01.

### ML models

Linear Regression^44^ aims to model the relationship between the dependent variable (AC Conductivity) and independent variables (Temperature, Frequency, Crystallite Size, Dislocation Density, Micro Strain, Permittivity) by fitting a linear equation:

21 $$\begin{aligned} \sigma _{AC} = \beta _0 + \beta _1 T + \beta _2 f + \beta _3 d + \beta _4 \delta + \beta _5 \epsilon _r + \epsilon \end{aligned}$$

where:

- $\sigma _{AC}$ is the AC conductivity,
- $T$ is the temperature,
- $f$ is the frequency,
- $d$ is the crystallite size,
- $\delta$ is the dislocation density,
- $\epsilon _r$ is the permittivity,
- $\epsilon$ is the error term,
- $\beta _i$ are the coefficients to be determined.

Random Forest^45^ is an ensemble learning method based on decision trees. It constructs multiple decision trees during training and outputs the mean prediction of the individual trees:

22 $$\begin{aligned} \sigma _{AC} = \frac{1}{N} \sum _{i=1}^{N} T_i(X) \end{aligned}$$

where:

- $N$ is the number of trees,
- $T_i(X)$ is the prediction of the $i$-th tree for the input features $X$.

Random Forest captures nonlinear relationships and interactions between features, making it robust for complex datasets.

Gradient Boosting^46^ is another ensemble technique that builds trees sequentially, each one correcting the errors of its predecessor. The model prediction is a weighted sum of the predictions from the individual trees:

23 $$\begin{aligned} \sigma _{AC} = \sum _{i=1}^{N} \alpha _i T_i(X) \end{aligned}$$

where:

- $N$ is the number of trees,
- $\alpha _i$ are the weights,
- $T_i(X)$ is the prediction of the $i$-th tree.

Gradient Boosting focuses on minimizing the residuals from the previous trees, thus improving the accuracy of predictions iteratively.

SVR^47^ aims to find a function that deviates from the actual observed values by a value no greater than $\epsilon$ for each training point while being as flat as possible. The function is given by:

24 $$\begin{aligned} f(X) = \sum _{i=1}^{l} (\alpha _i - \alpha _i^*) K(X_i, X) + b \end{aligned}$$

where:

- $K(X_i, X)$ is the kernel function (e.g., linear, polynomial, radial basis function),
- $\alpha _i, \alpha _i^*$ are the Lagrange multipliers,
- $b$ is the bias term.

SVR is effective for high-dimensional spaces and provides flexibility through the choice of different kernel functions. We used previous four ML algorithms to predict AC conductivity based on the dataset. Each model was trained and tested using standard performance metrics, including Root Mean Squared Error (RMSE), Mean Absolute Error (MAE), and $R^2$.

### Exploratory data analysis (EDA)

**Fig. 14:**
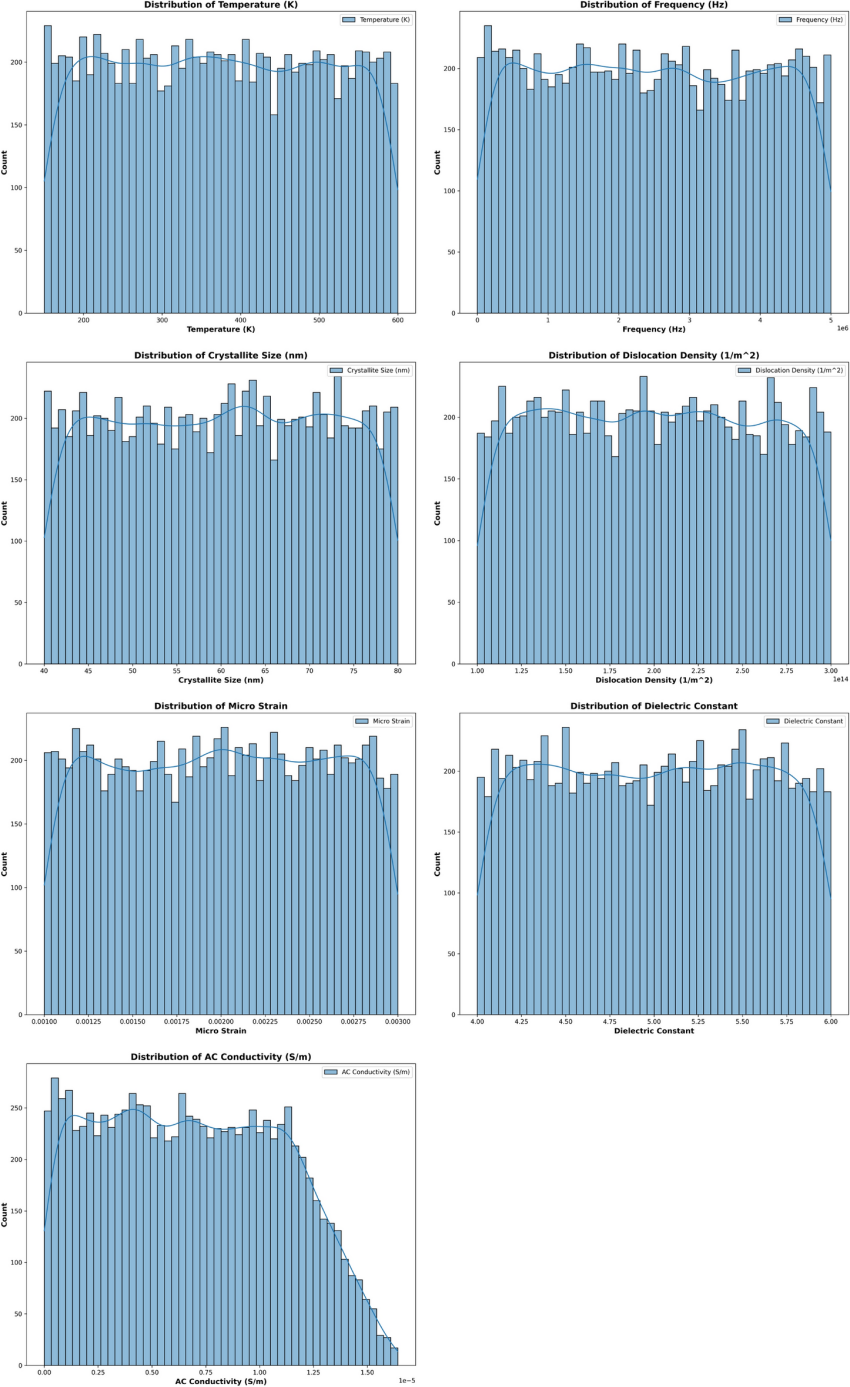
Histograms of features in synthetic dataset.

**Fig. 15:**
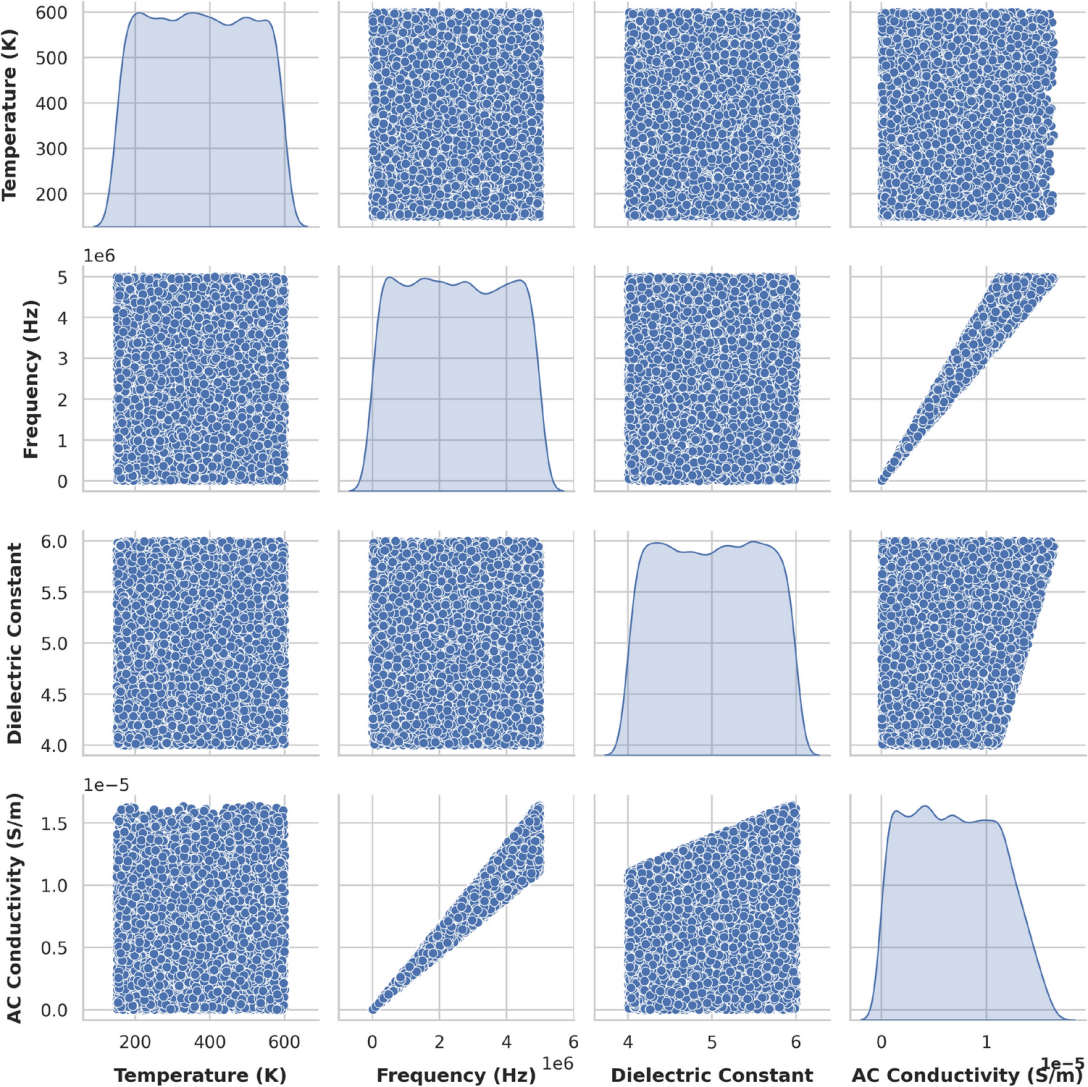
Pairplot of some features in synthetic dataset.

**Fig. 16:**
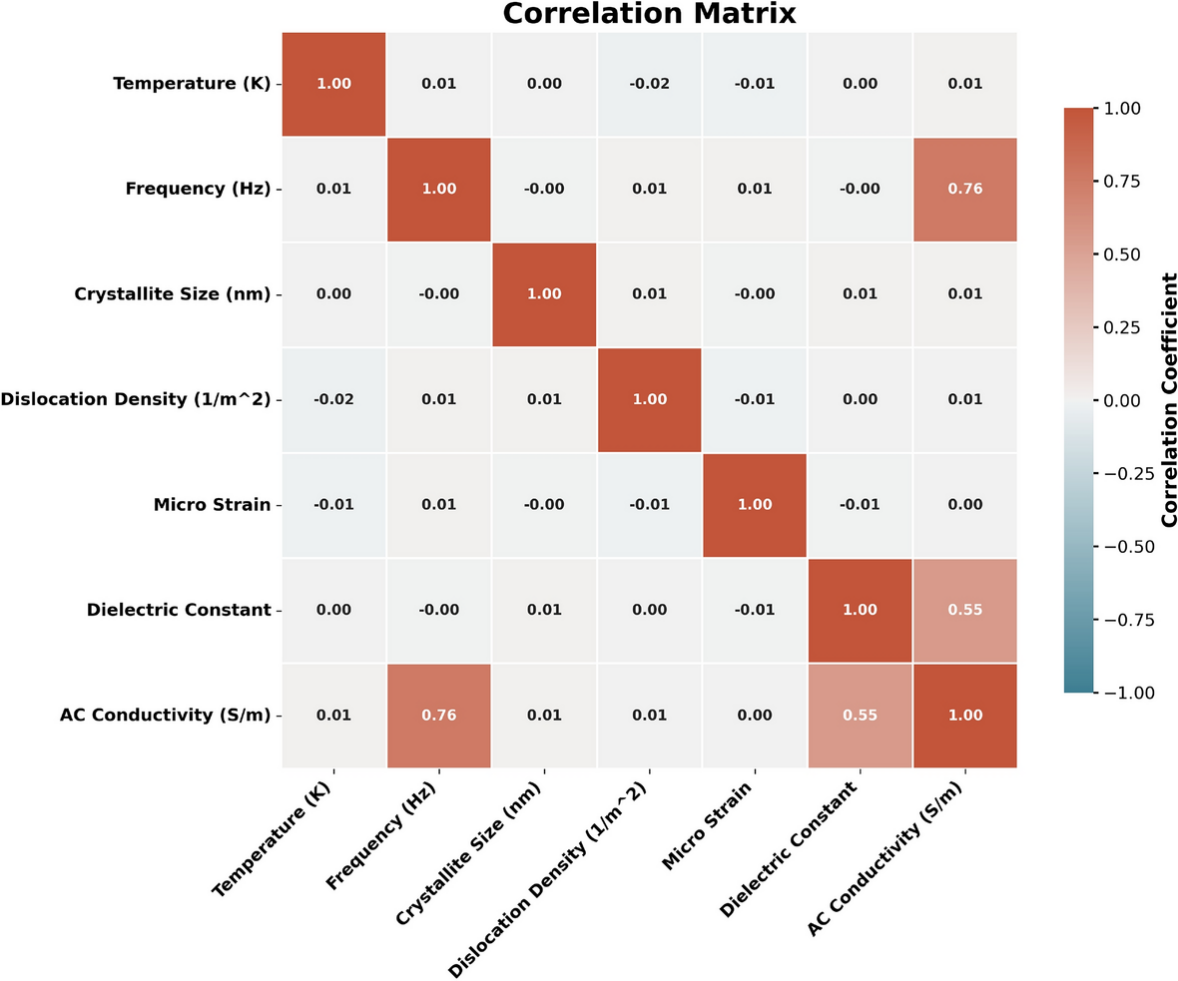
Correlation heatmap of features in synthetic dataset.

We analyzed the synthetic dataset to understand feature distributions and correlations. Figure 14 shows the histograms of individual features. Figures 15 and 16 display pairwise relationships and correlations, respectively. These visualizations highlight significant correlations, such as the strong relationship between frequency and AC conductivity. The summary statistics of the dataset are presented in Table 2. These statistics provide a numerical summary of the data, including measures of central tendency and variability.

**Table 2:** Summary statistics of synthetic dataset of 10k samples. The columns represent: Temp (Temperature in K), Freq (Frequency in Hz), Cryst Size (Crystallite Size in nm), Disl Dens (Dislocation Density in 1/m$^{2}$), Micro Strain, Dielect Const (Permittivity), and AC Cond (AC Conductivity in S/m).

	Temp (K)	Freq (Hz)	CSize (nm)	DD (1/m$^{2}$)	MS	Die const	ACC(S/m)
mean	373.41	$2.48 \times 10^6$	60.03	$1.99 \times 10^{14}$	0.0020	3.42	$4.70 \times 10^{-6}$
std	130.32	$1.46 \times 10^6$	11.57	$5.75 \times 10^{13}$	0.0006	1.48	$3.63 \times 10^{-6}$
min	150.03	833.21	40.01	$1.00 \times 10^{14}$	0.0010	0.85	$6.30 \times 10^{-10}$
25%	260.39	$1.21 \times 10^6$	49.96	$1.50 \times 10^{14}$	0.0015	2.12	$1.78 \times 10^{-6}$
50%	372.08	$2.47 \times 10^6$	60.23	$1.99 \times 10^{14}$	0.0020	3.44	$3.80 \times 10^{-6}$
75%	486.73	$3.75 \times 10^6$	70.06	$2.49 \times 10^{14}$	0.0025	4.70	$6.98 \times 10^{-6}$
max	599.99	$5.00 \times 10^6$	79.99	$3.00 \times 10^{14}$	0.0030	6.00	$1.62 \times 10^{-5}$

### Model performance and results

Table 3 summarizes the performance of the ML models. Random Forest and Gradient Boosting achieved the highest accuracy, with $R^2$ values of 0.9998 and 0.9987, respectively. Linear Regression performed well for linear trends but lacked precision for nonlinear dependencies. SVR performed poorly, with a negative $R^2$, indicating it is unsuitable for this dataset.

**Table 3:** Performance metrics for ML models.

Model	RMSE	MAE	$R^2$
Linear Regression	0.0000	0.0000	0.8909
Random Forest	0.0000	0.0000	0.9998
Gradient Boosting	0.0000	0.0000	0.9987
SVR	0.0000	0.0000	-0.7986

Feature importance analysis revealed that temperature and frequency are the most critical factors influencing AC conductivity. These findings agree with experimental results, validating the CBH model.

### Integration of ML and experimental findings

The results confirm a strong alignment between ML predictions and experimental data. Random Forest and Gradient Boosting accurately modeled AC conductivity, reflecting the experimental observations. This agreement validates the CBH model and highlights the potential of ML to enhance understanding of charge transport mechanisms. By combining ML insights with lab experiments, we identified optimal conditions for synthesizing $\text {TlInTe}_2$ crystals with desired electrical properties. This interdisciplinary approach bridges computational and experimental techniques, advancing the design and development of high-performance semiconductor materials.

## Conclusions

TlInTe$_2$ in powder form, prepared from cleaved single crystals, was confirmed to crystallize in a tetragonal system at room temperature. Structural parameters such as crystallite size (*D*), dislocation density ($\delta$), and micro strain ($\epsilon$) were derived from XRD spectra. Thermo-gravimetric analysis (TGA) revealed excellent thermal stability over a wide temperature range, making TlInTe$_2$ a strong candidate for semiconductor applications. Future research should focus on refining synthesis techniques to further enhance its thermal stability and expand its potential for high-temperature applications.

The frequency and temperature dependence of AC conductivity validated the applicability of the CBH model for bulk TlInTe$_2$. The smaller activation energy values and the temperature-dependent behavior of the frequency exponent (*s*) strongly support this conclusion. The AC conductivity $\sigma _{\text {ac}}(\omega )$ is a thermally activated process, consistent with the CBH model. Key parameters such as the density of localized states at the Fermi level $N(E_F)$, average hopping time *t*, and hopping distance *R* were determined to be in the ranges of $1.02-2.8 \times 10^{19}\,\text {eV}^{-1}\,\text {cm}^{-3}$, $2 \times 10^{-7}-2.4 \times 10^{-2}\,\hbox {s}$, and $6.10{-}11.95\,\hbox {nm}$, respectively. Additionally, the maximum barrier height $W_m$ was calculated to be 0.52 eV for bulk TlInTe$_2$. The absence of anomalies in the permittivity across the studied temperature range (294–393 K) suggests no phase transitions occur within this range. The dielectric relaxation mechanism was explained using the complex dielectric modulus, where the temperature dependence of molecular relaxation time followed a thermally activated process.

ML played a critical role in augmenting the experimental analysis. Predictive modeling using ensemble learning techniques, such as Random Forest and Gradient Boosting, provided additional insights into the electrical properties of TlInTe$_2$. These models identified key predictors of AC conductivity and computationally validated the CBH model. The congruence between ML predictions and experimental findings confirmed the reliability of the data and demonstrated the potential of ML to enhance our understanding of material properties. The statistical significance tests highlighted that Random Forest slightly outperformed Gradient Boosting in predictive accuracy.

This integration of experimental and computational approaches highlights the potential of ML to complement traditional experimental techniques. It opens up new possibilities for predictive materials science, where computational tools accelerate the discovery and optimization of novel semiconductor materials. The findings in this study demonstrate a robust framework for combining experimental validation with computational analysis to explore and optimize material properties.

## Data Availability

The datasets generated and analyzed during the current study are available from the corresponding author on reasonable request.
